# Exploring the Significance of Immune Checkpoints and EBV Reactivation in Antibody Deficiencies with Near-Normal Immunoglobulin Levels or Hyperimmunoglobulinemia

**DOI:** 10.3390/cancers15205059

**Published:** 2023-10-19

**Authors:** Paulina Mertowska, Sebastian Mertowski, Konrad Smolak, Marcin Pasiarski, Jolanta Smok-Kalwat, Stanisław Góźdź, Ewelina Grywalska

**Affiliations:** 1 Department of Experimental Immunology, Medical University of Lublin, 20-093 Lublin, Poland; paulinamertowska@umlub.pl (P.M.); ewelina.grywalska@umlub.pl (E.G.); 2 Department of Immunology, Faculty of Health Sciences, Jan Kochanowski University, 25-317 Kielce, Poland; marcinpasiarski@gmail.com; 3 Department of Hematology, Holy Cross Cancer Centre, 25-734 Kielce, Poland; jolantasmok1@gmail.com (J.S.-K.); stanislawgozdz1@gmail.com (S.G.); 4 Institute of Medical Science, Collegium Medicum, Jan Kochanowski University of Kielce, IX Wieków Kielc 19A, 25-317 Kielce, Poland

**Keywords:** EBV, immune system, immune checkpoint, PD-1, PD-L1, CTLA-4, CD86, CD200R, CD200, immunodeficiency, cancer risk

## Abstract

**Simple Summary:**

This article addresses the topic of primary immunodeficiencies, with particular emphasis on antibody deficiencies with near-normal immunoglobulin levels or hyperimmunoglobulinemia. This paper goes beyond genetics and emphasizes the importance of the immune system and particularly immune checkpoints and Epstein–Barr virus (EBV) reactivation in the context of these disorders. The article delves into the immune dysregulations occurring in the course of this type of disease and the potential role of EBV reactivation, which affects the clinical picture of patients and, in the future, may contribute to the development of cancer, especially those related to hematological malignancies. Disturbances observed in the immunopathogenesis of the presented diseases go beyond the accepted scheme, with the development of PID largely associated only with genetic disorders, and the article emphasizes that the regulation of immunity and virus reactivation also contributes to the progression of PID.

**Abstract:**

This study delves into the intricate landscape of primary immunodeficiencies, with a particular focus on antibody deficiencies characterized by near-normal immunoglobulin levels or hyperimmunoglobulinemia. Contrary to the conventional focus on genetic dysregulation, these studies investigate the key roles of immune checkpoints, such as PD-1/PD-L1, CTLA-4/CD86, and CD200R/CD200, on selected subpopulations of T and B lymphocytes and their serum concentrations of soluble forms in patients recruited for the studies in healthy volunteers. In addition, the studies also show the role of Epstein–Barr virus (EBV) reactivation and interactions with tested pathways of immune checkpoints involved in the immunopathogenesis of this disease. By examining the context of antibody deficiencies, this study sheds light on the nuanced interplay of factors beyond genetics, particularly the immune dysregulations that occur in the course of this type of disease and the potential role of EBV reactivation, which affects the clinical presentation of patients and may contribute to the development of cancer in the future, especially related to hematological malignancies.

## 1. Introduction

Primary immunodeficiencies (PIDs) are genetic diseases that affect the ability of the immune system to function properly. They can involve various components of the immune response, including T cells, B cells, phagocytes, and others. Currently, more than 300 different immunodeficiencies are known. One such example consists of antibody deficiencies with near-normal immunoglobulin levels or hyperimmunoglobulinemia [1,2,3,4].

Antibody deficiency refers to a condition in which a person’s immune system is unable to produce enough antibodies, which are crucial to fighting infection [5,6]. This deficiency means that, although the number of antibodies is adequate, their quality or ability to effectively fight infection may be compromised. This compromise can be due to problems such as B cell dysfunction, impaired antibody maturation, or defects in the interactions between immune cells [5,7,8]. Hyperimmunoglobulinemia refers to the presence of abnormally high levels of immunoglobulins in the blood. Although it may seem counterintuitive, hyperimmunoglobulinemia can also be associated with certain antibody deficiencies. In these cases, even if there is an excess of antibodies, the antibodies may not be able to effectively fight infection due to functional problems [9,10,11,12].

People with this type of immunodeficiency have relatively normal levels of immunoglobulins (IgG, IgA, and IgM) but show impaired antibody responses to specific antigens (foreign substances that trigger an immune response). Some key clinical findings include recurrent infections (bacterial infections, especially in the respiratory and gastrointestinal tracts), normal immune cell counts (total immune cell counts, whether T cells or B cells, may be within the normal range), and decreased antibody responses (a person’s immune system does not produce enough antibodies when exposed to certain antigens, such as those present in vaccines or certain pathogens) [13,14,15,16].

An antibody deficiency with near-normal immunoglobulin levels or hyperimmunoglobulinemia may be associated with an increased risk of certain health complications, including an increased risk of developing certain types of cancer. This cancer risk is often caused by chronic or severe infections, which over time can potentially contribute to the development of some cancers, including breast cancer, and cardiovascular cancers, such as multiple myeloma, leukemia, and lymphoma. Persistent inflammation and dysregulation of the immune system associated with chronic infections can further create an environment conducive to tumor growth [14,16,17,18,19].

Increasing studies in the literature have also indicated the involvement of the Epstein–Barr virus (EBV) in the development of neoplastic diseases in the course of PID [20,21]. This virus is a common herpes virus that infects a large proportion of the world’s population. While most people infected with EBV experience mild or no symptoms, the virus can cause infectious mononucleosis and is associated with a variety of conditions, including certain types of cancer. The relationship between EBV and PID is complex, and its impact on people with these disorders, as researchers have emphasized, may vary from exacerbated clinical symptoms through increased deregulation of the immune system to the development of cancer [22,23].

Immune checkpoints, which are regulatory molecules crucial to maintaining immune balance, have gained attention for their involvement in a variety of immune-related disorders, including cancer and autoimmune diseases [24,25,26]. These checkpoints serve as modulators of immune responses, affecting the activation, proliferation, and function of immune cells [27,28,29,30]. In antibody deficiencies, dysregulated immune checkpoint expression may contribute to the insufficient antibody production observed despite near-normal immunoglobulin levels or hyperimmunoglobulinemia [31].

Moreover, immune checkpoints can influence the immune response to pathogens, such as Epstein–Barr virus (EBV). EBV reactivation is of particular importance in these disorders due to the impaired immune response. Dysfunctional immune checkpoints may contribute to the inability to effectively control EBV reactivation, leading to recurrent infections and potential complications. Understanding the intricate relationships among immune checkpoints, antibody deficiencies, and EBV reactivation is essential for the development of targeted therapeutic approaches [32,33].

Therefore, this study aimed to assess the percentage of T and B lymphocytes positively expressing three immune checkpoint pathways, namely PD-1/PD,-L1, CTLA-4/CD86, and CD200R/CD200, and the concentrations of their soluble forms in the serum of patients with antibody deficiencies with near-normal immunoglobulin levels or hyperimmunoglobulinemia compared to healthy volunteer controls. Moreover, we extended our research by assessing the occurrence of EBV reactivation based on serological profiles of antibodies against specific antigens of this virus, as well as assessing the number of its copies in the tested genetic material.

We hope that the research results presented in this manuscript will make it possible to draw special attention to the role of immune checkpoints in the immunopathogenesis of the described disease and the potential relationship with reactivation of the oncogenic EBV virus in the bodies of patients, which may contribute to an increased risk of hematological cancers.

## 2. Materials and Methods

### 2.1. Patient Characteristics

The study group consisted of 40 patients diagnosed with antibody deficiencies with near-normal immunoglobulin levels or hyperimmunoglobulinemia and 20 healthy volunteers as a control group. The median age of patients with antibody deficiencies with near-normal immunoglobulin levels or hyperimmunoglobulinemia was 41.00 years old (range: 26.00–56.00), and that of the healthy volunteers was 42.00 (range: 18.00–67.00). All patients were age matched and subject to inclusion and exclusion criteria. Individuals who met any of the following exclusion criteria were not included in the study:

a. Having an ongoing viral, bacterial, or fungal infection;
b. Suffering from severe allergies;
c. Having a history of hematopoietic cell or organ allotransplantation;
d. Undergoing treatment for an active malignancy or any other autoimmune disease;
e. Being pregnant or lactating;
f. Using investigational drugs;
g. Having tumor metastases in the central nervous system or mental illness.

The adopted patient selection criteria were aimed at eliminating most interfering factors that could negatively affect the functioning of the immune system and influence the obtained test results. Moreover, the selection of patients according to age allowed us to eliminate factors related to the patients’ ages, which could negatively affect the obtained test results. All patients included in this study were newly diagnosed, meaning that they were not yet subject to any therapies or medications that could negatively affect the obtained test results. Participants from both the study group and the control group came from the Świętokrzyskie voivodeship. Recruitment of patients for this study was performed by a physician specializing in clinical immunology. Patients from the control group were healthy people matched to the study group, and they were also subject to the same inclusion and exclusion criteria as patients from the study group. The research material consisted of 10 mL of peripheral blood collected in EDTA tubes (used for morphology and biochemistry analysis and immunophenotyping, as well as isolation of peripheral blood mononuclear cells (PBMCs) to obtain genetic material for determining the number of virus copies) and 5 mL of blood collected for a clot sample in to obtain serum (to determine the concentrations of soluble forms of the test molecules and to assess EBV reactivation based on serological profiles).

### 2.2. Quantification of EBV Genomic Copies in PBMC-Derived DNA

The quantification of Epstein–Barr virus (EBV) genomic copies was carried out utilizing the ISEX variant of the EBV PCR assay (GeneProof, Brno, Czech Republic). According to the manufacturer’s information, the diagnostic specificity is 94.19% (95% CI: 86.35–97.84%), and the sensitivity is 99.10% (95% CI: 96.45–99.84%). The range of detection of the number of copies of the EBV virus in the tested genetic material is greater than 10 copies. To ensure precision, duplicate assessments were conducted on all samples, and a negative control containing DNA elution buffer was included. Amplification was targeted at the conserved DNA sequence specific to the *EBNA1* gene of EBV, employing the 7300 Real-Time PCR System (Applied Biosystems, Foster City, CA, USA). The process was strictly conducted in accordance with the ISEX EBV PCR kit protocol. The final concentration of viral DNA copies, expressed per microgram of extracted DNA, was normalized, considering the DNA isolation efficiency. A detection threshold was established at 10 EBV DNA copies/μL, and samples less than this level were deemed EBV-negative.

### 2.3. Serological Profiling of Anti-EBV Specific Antibodies

We executed a qualitative analysis to detect the presence of specific IgA, IgM, and IgG antibodies targeted against selected Epstein–Barr virus (EBV) antigens, namely viral-capsid antigen (VCA), early antigen (EA), and Epstein–Barr nuclear antigen 1 (EBNA1), in the sera of both study and control cohorts. We leveraged commercial ELISA kits designed to discern antibodies of classes IgA, IgG, and IgM specific to these EBV antigens. The protocols followed the stipulated guidelines of the kit manufacturer. The assay employed a suite of kits: EBV VCA IgA, EBV EA IgA, EBV EBNA1 IgA, EBV VCA IgG, EBV EA IgG, EBV EBNA1 IgG, EBV VCA IgM, EBV EA IgM, and EBV EBNA1 IgM (Demeditec Diagnostics GmbH, Kiel, Germany). Absorbance readings were captured using the VictorTM3 microplate reader (PerkinElmer, Waltham, MA, USA). Antibody titers, quantified from these readings, are represented in U/mL, adhering to the calibration guidelines provided by the manufacturer. A titer value surpassing 11 was designated as positive.

### 2.4. Assessment of Soluble Immune Checkpoint and Ligand Concentrations in Serum

The soluble immune checkpoint and ligand concentrations in serum were assessed by conducting immunoenzymatic assays using serum samples collected from all participants. We used commercial kits for this purpose, as follows: human CD200 ELISA Kit (sensitivity: 20 pg/mL) (Invitrogen, Waltham, MA, USA); human CD200R ELISA Kit (sensitivity: 11.89 pg/mL) (Abcam, Cambridge, UK); human CTLA-4 ELISA Kit (Sensitivity: 0.13 ng/mL)(Invitrogen, Waltham, MA, USA); human CD86 ELISA Kit (Sensitivity: 0.82 ng/mL) (Invitrogen, Waltham, MA, USA); human PD-1 ELISA Kit (Sensitivity: 1.14 pg/mL) (Invitrogen, Waltham, MA, USA); and human PD-L1 ELISA Kit (Sensitivity: 0.6 pg/mL) (Invitrogen, Waltham, MA, USA). We measured the concentration of all tested molecules using a VictorTM3 reader (PerkinElmer, Waltham, MA, USA). The manufacturers’ instructions were followed when using the kits.

### 2.5. Lymphocyte Immunophenotyping

Using flow cytometry, blood samples were processed with an extensive panel of human-specific monoclonal antibodies. These antibodies included anti-CD3 PerCp, anti-CD4 BV421, anti-CD8 BV605, anti-CD19 FITC, and anti-CD45 AF700, along with other antibodies targeting immune checkpoints and pertinent markers, such as anti-PD-1 APC, anti-PD-L1 PE, anti-CTLA-4 PE, anti-CD86 APC, anti-CD200 PE, and anti-CD200R APC. All of these antibodies were sourced from BioLegend (San Diego, CA, USA). For accurate gating during cytometric analysis, FMO controls were incorporated specifically for the immune checkpoints’ antibodies.

**Figure 1:**
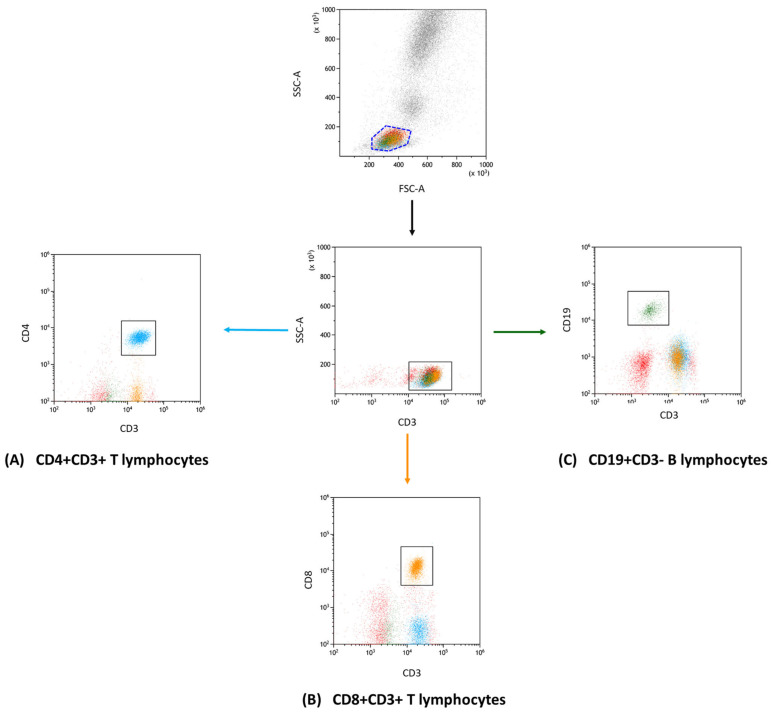
Flow cytometry gating and analysis strategy. This diagram showcases the gating approach that we used in our research to define lymphocyte subgroups. In detail, the subsets presented are (**A**) CD4+ CD3+ (indicated in blue); (**B**) CD8+ CD3+ (marked in orange); and (**C**) CD19+ CD3− (displayed in green). This categorization enabled the further investigation of unique immune checkpoint markers within each group.

**Figure 2:**
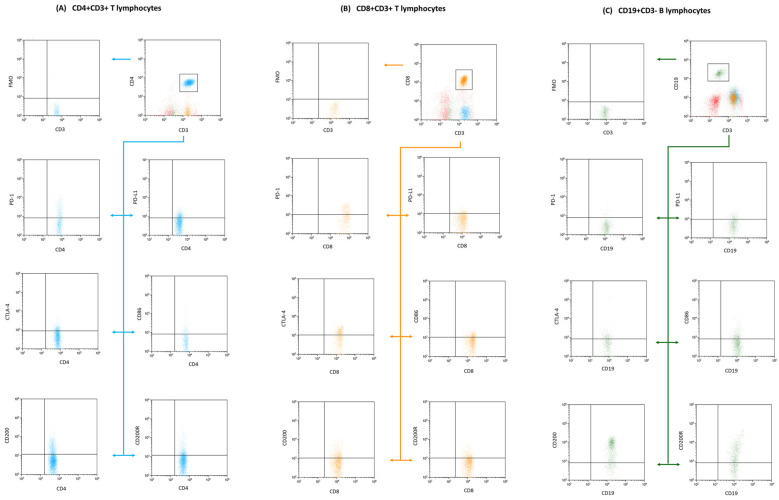
Flow cytometry segmentation and analysis outline: The displayed diagram underscores our gating methods used to isolate T and B lymphocyte clusters. We focused on phenotypic expressions, of CD4+ CD3+ T lymphocytes subfigure (**A**), (denoted in blue); CD8+ CD3+ T lymphocytes subfigure (**B**), (tagged in orange), and CD19+ CD3− B lymphocytes subfigure (**C**), (depicted in green). These clusters were subsequently probed for markers including CD200, CD200R, CTLA-4, CD86, PD-1, and PD-L1. To ensure precision in our gating and analysis, fluorescence minus one (FMO) controls were integrated, providing a clear comparative baseline to fine-tune our gating settings.

After the antibody incubation stage, red blood cells were lysed using a specific buffer from BD (Franklin Lakes, NJ, USA). This lysing solution was prepared following the manufacturer’s protocol to ensure optimal cell lysis. The post-lysis cell suspension was then washed and analyzed on the CytoFLEX LX cytometer (Beckman Coulter, Indianapolis, IN, USA). For subsequent data interpretation, Kaluza Analysis software, version 2.1, also from Beckman Coulter, was used. The CytoFLEX LX system’s consistent quality was upheld using CytoFLEX Ready to Use Daily QC Fluorospheres reagents (Beckman Coulter, Indianapolis, IN, USA). Figure 1 and Figure 2 present the results of the sample analysis.

### 2.6. Statistical Analysis of Obtained Results

The statistical analysis of the collected data was performed using Tibco Statistica software, version 13.3 (Palo Alto, CA, USA). The normality of the data distribution was evaluated through the application of the Shapiro–Wilk test. The Kruskal–Wallis test was utilized to analyze differences between groups, followed by Dunn’s post hoc test. The *p*-values for Dunn’s test were adjusted using Bonferroni’s method to account for multiple comparisons. Spearman’s correlation coefficients were employed to investigate the relationships between pairs of variables. ROC curves were generated to determine the diagnostic performance of laboratory tests for patient-related parameters. GraphPad Prism software, version 9.4.1 (San Diego, CA, USA), was used to visualize the data (especially the graphs presented in this publication).

## 3. Results

### 3.1. Analysis of the History and Basic Clinical Parameters of Patients with Antibody Deficiencies with Near-Normal Immunoglobulin Levels or Hyperimmunoglobulinemia

The first stage of our research was to conduct comparative analyses of selected aspects of the disease history (number of infections requiring antibiotic therapy) and parameters of morphology and peripheral blood biochemistry of patients from the study group and healthy volunteers. The suspicion of PID is usually related to the occurrence of infections characterized by exceptional frequency or severity, lack of appropriate response to the forms of treatment used, and unusual types of bacteria, viruses, or fungi causing them. The most frequently observed infections in adults with primary immunodeficiency are respiratory infections, including infections of the paranasal sinuses, lower respiratory tract (bronchitis), or lungs, as well as digestive problems (causing diarrhea). Based on the collected medical history, we determined that all patients included in the study had numerous infections requiring antibiotic therapy before the diagnosis. The number of recorded infections in the previous year before diagnosis ranged from 6 to 9 in 32.5% of patients, between 10 and 12 infections in 52.5% of patients, and more than 12 infections in 15% of patients. Moreover, a detailed analysis showed that most infections were related to the respiratory tract (100% of patients), gastrointestinal tract (60% of patients), urinary tract (40% of patients), and skin (30% of patients). Additionally, only 10 patients had a family history of PID. None of the patients included in the study were diagnosed with allergies or signs of autoimmunity, as well as comorbidities. The analysis of selected test results, including complete blood count and biochemistry, showed no IgA and IgM deficiencies, as well as no signs of leukopenia or thrombocytopenia. Detailed research data are presented in Table 1.

We also assessed selected peripheral blood immunophenotype parameters, suggesting the state of the patients’ immune systems at the time of their recruitment, i.e., at the time of diagnosis. Here, we observed much more statistically significant differences between the study group and healthy volunteers. These changes included a decrease in the percentage of CD3+ T cells, accompanied by a decrease in the percentage of CD4+ T cells (no change in CD8+ T cells), a decrease in the ratio of CD4+ T cells to CD8+ T cells, and a decrease in the percentage of CD19+ B cells (Table 2).

### 3.2. Evaluation of the Expression of PD-1/PD-L1, CTLA-4/CD86, and CD200R/CD200 on T and B Lymphocytes in Patients with Antibody Deficiencies with Near-Normal Immunoglobulin Levels or Hyperimmunoglobulinemia in Relation to Healthy Volunteers

**Figure 3:**
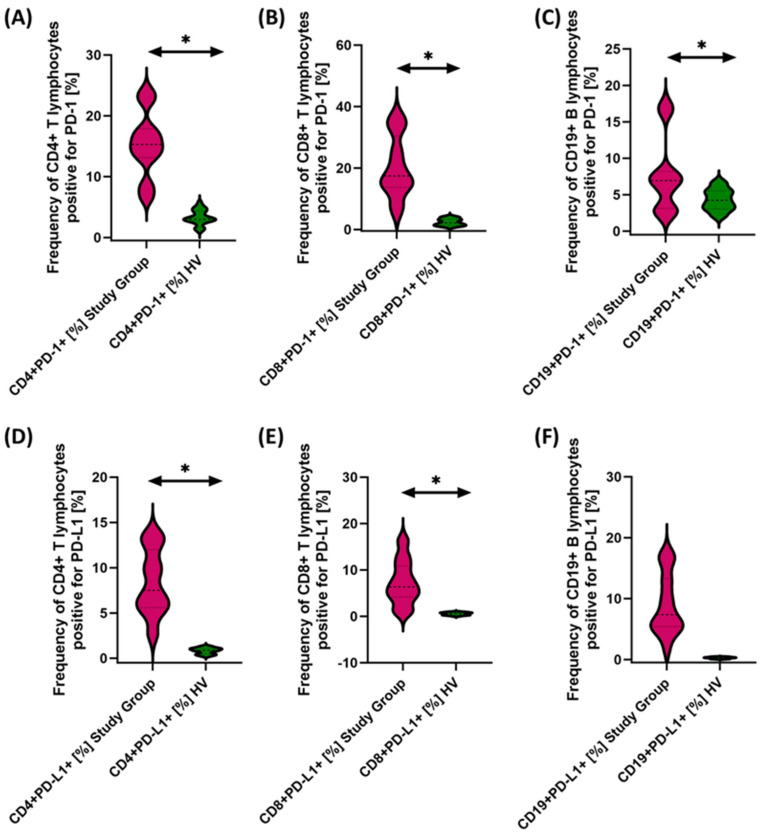
Evaluation of the percentages of T and B lymphocytes positively expressing PD-1 (**A**–**C**) and PD-L1 (**D**–**F**) in patients with antibody deficiencies with near-normal immunoglobulin levels or hyperimmunoglobulinemia relative to healthy volunteers. Statistically significant results are marked with *. Patients with antibody deficiencies with near-normal immunoglobulin levels or hyperimmunoglobulinemia are marked in pink, and the group of healthy volunteers without EBV reactivation is marked in green.

**Figure 4:**
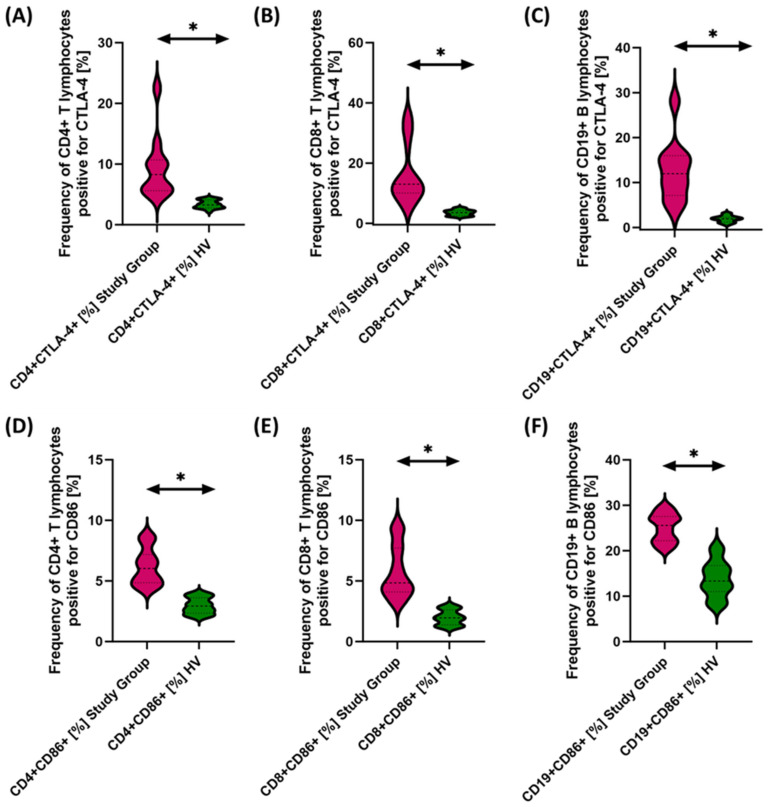
Evaluation of the percentages of T and B cells positive for CTLA-4 (**A**–**C**) and CD86 (**D**–**F**) expression in antibody deficiencies with near-normal immunoglobulin levels or hyperimmunoglobulinemia relative to healthy volunteers. Statistically significant results are marked with *. Pink indicates patients with antibody deficiencies with near-normal immunoglobulin levels or hyperimmunoglobulinemia, and green indicates the group of healthy volunteers without EBV reactivation.

**Figure 5:**
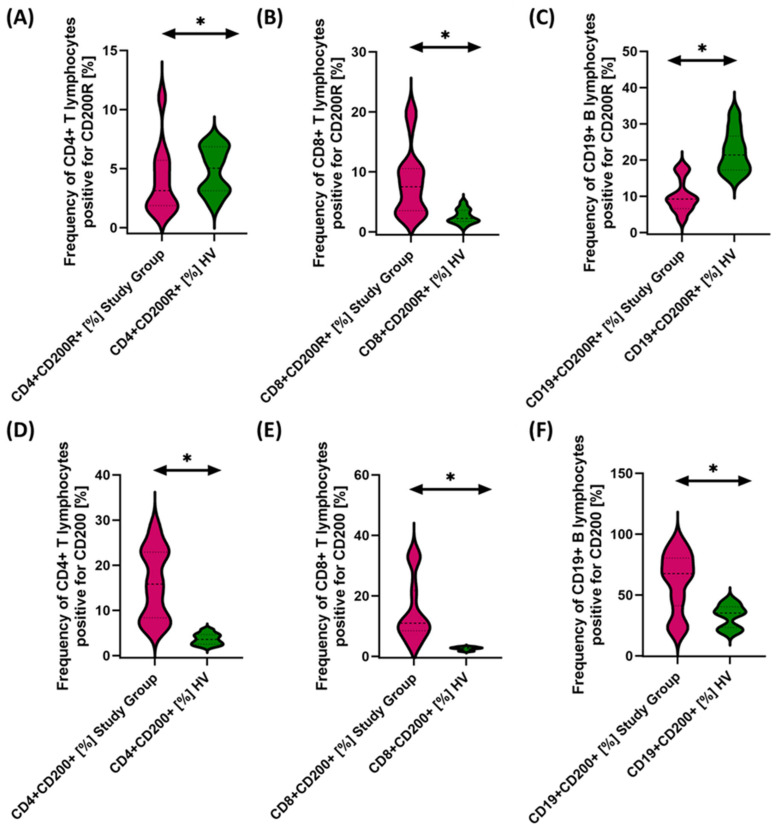
Evaluation of the percentages of T and B cells positively expressing CD200R (**A**–**C**) and CD200 (**D**–**F**) in patients with antibody deficiencies with near-normal immunoglobulin levels or hyperimmunoglobulinemia relative to healthy volunteers. Statistically significant results are marked with *. Pink indicates patients with antibody deficiencies with near-normal immunoglobulin levels or hyperimmunoglobulinemia, and green indicates the group of healthy volunteers without EBV reactivation.

Due to the changes in the percentage of selected immune cells observed in the immunophenotype, in the next stage of the research, we conducted an assessment of PD-1/PD-L1, CTLA-4/CD86, and CD200R/CD200 in selected subpopulations of T and B lymphocytes in patients with antibody deficiencies with near-normal immunoglobulin levels or hyperimmunoglobulinemia in relation to healthy volunteers. Detailed test data are presented in Table 3 and illustrated in Figure 3, Figure 4 and Figure 5.

The studies showed a statistically significant increase in almost all tested immune checkpoints and their ligands regarding selected subpopulations of T and B lymphocytes in patients with antibody deficiencies with near-normal immunoglobulin levels or hyperimmunoglobulinemia relative to healthy volunteers (Figure 3A–F, Figure 4A–F and Figure 5B,D–F), except for the percentages of CD4+ CD200R+ and CD19+ CD200R (Figure 5A,C), which were higher in healthy volunteers.

### 3.3. Evaluation of PD-1/PD-L1, CTLA-4/CD86, and CD200R/CD200 Concentrations in the Serum of Patients with Antibody Deficiencies with Near-Normal Immunoglobulin Levels or Hyperimmunoglobulinemia in Relation to Healthy Volunteers

**Figure 6:**
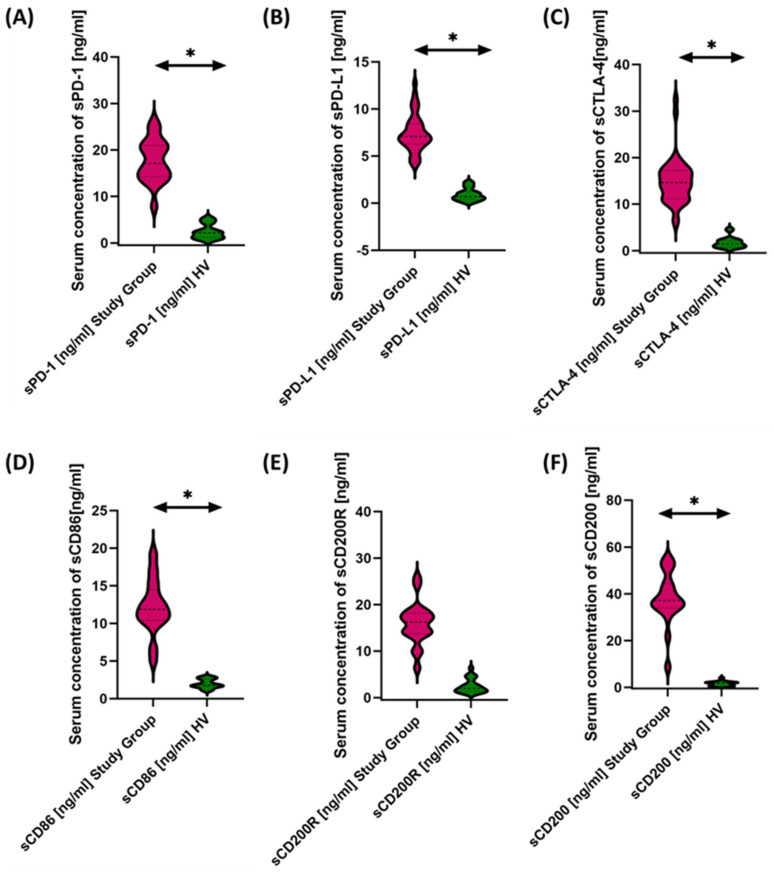
Serum concentrations of PD-1/PD-L1 (**A**,**B**), CTLA-4/CD86 (**C**,**D**), and CD200R/CD200 (**E**,**F**) in antibody deficiencies with near-normal immunoglobulin levels or hyperimmunoglobulinemia compared to healthy volunteers. Statistically significant results are marked with *. Pink indicates the group of patients with antibody deficiencies with near-normal immunoglobulin levels or hyperimmunoglobulinemia, and green indicates the group of healthy volunteers.

We then evaluated the serum concentrations of the test molecules in patients with antibody deficiencies with near-normal immunoglobulin levels or hyperimmunoglobulinemia relative to healthy volunteers, and the results obtained are illustrated in Figure 6 and Table 4.

Based on the obtained data, we can conclude that, in patients with antibody deficiencies with near-normal immunoglobulin levels or hyperimmunoglobulinemia, we observed significantly elevated levels of all tested molecules in serum compared to healthy volunteers (Figure 6A–F).

### 3.4. Effect of EBV Reactivation on the Tested Pathways of Immune Checkpoints in the Course of Antibody Deficiencies with Near-Normal Immunoglobulin Levels or Hyperimmunoglobulinemia

**Figure 7:**
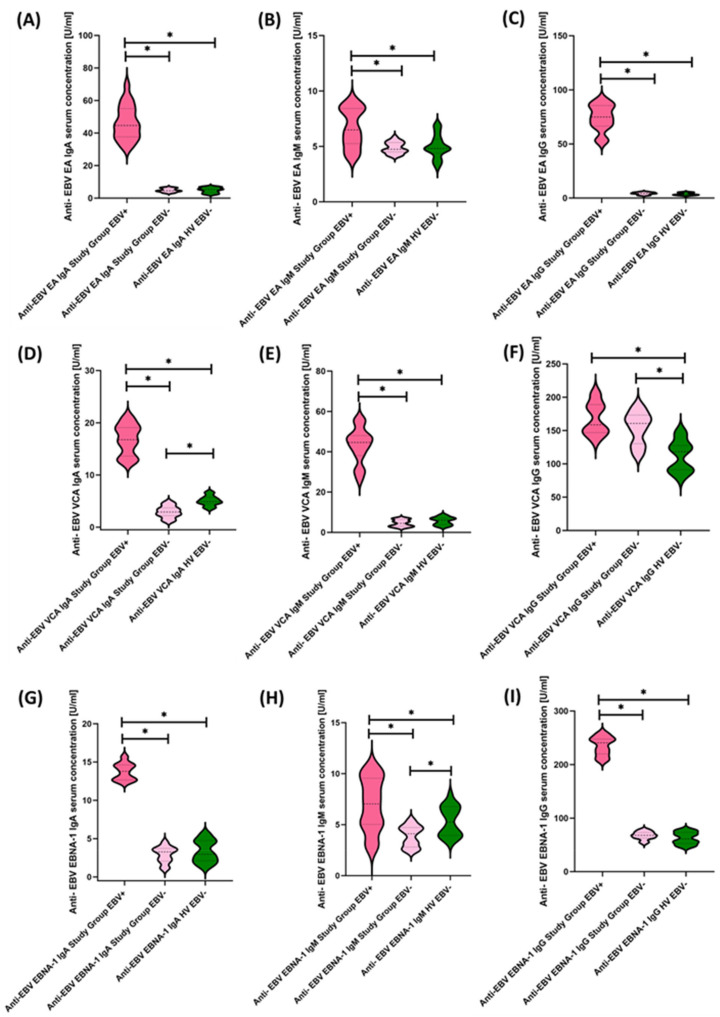
Evaluation of serological profiles related to EBV reactivation based on the concentrations of antibodies: anti-EBV EA in the class IgA (**A**), IgM (**B**), and IgG (**C**); anti-EBV VCA in IgA (**D**), IgM (**E**), and IgG (**F**) classes; anti-EBV EBNA-1 in IgA (**G**), IgM (**H**), and IgG (**I**) classes. Statistically significant results are marked with *. Dark pink represents patients with antibody deficiencies with near-normal immunoglobulin levels or hyperimmunoglobulinemia showing EBV reactivation, light pink represents patients with antibody deficiencies with near-normal immunoglobulin levels or hyperimmunoglobulinemia without EBV reactivation, and green indicates the group of healthy volunteers without EBV reactivation.

In the next stage of the research, we decided to check how EBV affects the obtained test results and more precisely whether and how it is reactivated and what changes it causes at the level of the immune system. Therefore, the first step was to assess serum antibody concentrations against specific EBV antigens, such as EA, VCA, and EBNA-1. The test results obtained from this experiment are shown in Figure 7 and Table 5.

To our surprise, 50% of the tested population showed EBV reactivations, which allowed us to divide the study patients into three equal groups: Study Group EBV+, Study Group EBV−, and Healthy Volunteers EBV−. Moreover, the EBV copy count determined with a commercial kit showed that reactivating patients had a mean value of 543.46 ± 161.95 (214.46–788.73) in test detection, i.e., fewer than 10 copies.

In the next stages of our analyses, we decided to check how the previously described clinical parameters changed, including the numbers and types of infections, changes in the morphology and biochemistry of peripheral blood, and immunophenotypic parameters.

The first aspect concerned data from the medical and family histories. In EBV+ patients, the number of infections was, respectively, greater than 12 in 25%, between 10 and 12 in 65%, and between 6 and 9 in 10% of patients. In the case of EBV− patients, the number of infections in the year preceding the diagnosis was greater than 12 in 5%, between 10 and 12 in 40%, and between 6 and 9 in 55% of patients. This finding means that EBV+ patients had more infections than EBV− patients. Another analyzed aspect was the type of observed infections, and in patients with EBV+, the most common infections were respiratory tract infections, which occurred in 100% of patients, followed by gastrointestinal infections, which were observed in 60% of patients, rut skin infections in 40% of patients, and finally urinary tract infections affecting 20% of patients. In analyses of pooled data in the context of EBV− patients, respiratory tract infections were observed in 90% of patients, urinary and gastrointestinal infections in 60% of patients, and skin infections in 20% of patients. Family history analysis also showed that 20% of EBV+ patients had a family history of PID, while 30% of EBV− patients had a family history. We also did not observe statistically significant differences between the age of the analyzed patients and healthy volunteers, as well as between patients with antibody deficiencies with near-normal immunoglobulin levels or hyperimmunoglobulinemia EBV+ and EBV−.

The analysis of peripheral blood morphology and biochemistry parameters, as well as immunophenotypic evaluation, also showed a number of significant changes between EBV+ and EBV− patients and healthy volunteers. The obtained results are presented in Table 6.

**Figure 8:**
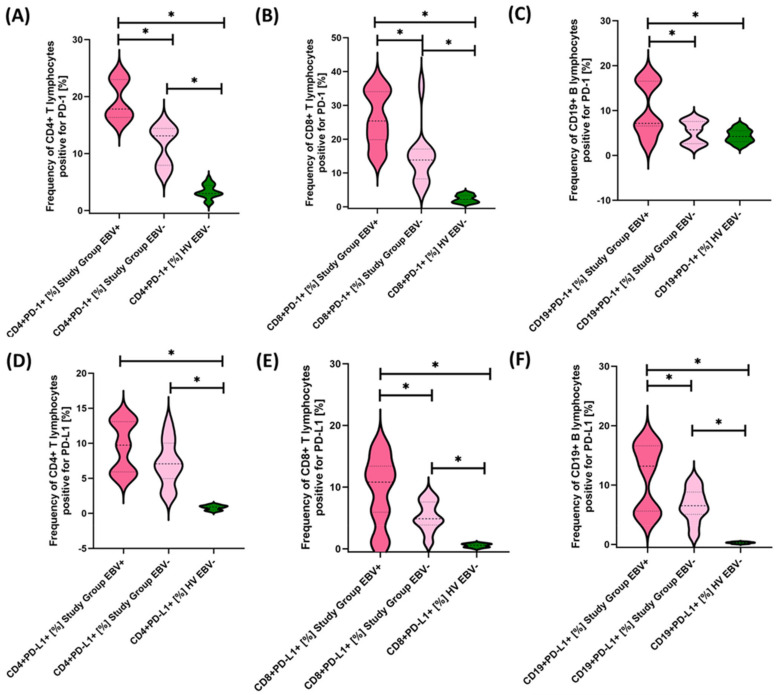
Evaluation of the percentages of T and B lymphocytes positive for PD-1 (**A**–**C**) and PD-L1 (**D**–**F**) expression in patients with antibody deficiencies with near-normal immunoglobulin levels or hyperimmunoglobulinemia, considering EBV reactivation relative to healthy volunteers. Statistically significant results are marked with *. Dark pink represents patients with antibody deficiencies with near-normal immunoglobulin levels or hyperimmunoglobulinemia showing EBV reactivation, light pink represents patients with antibody deficiencies with near-normal immunoglobulin levels or hyperimmunoglobulinemia without EBV reactivation, and green indicates the group of healthy volunteers without EBV reactivation.

**Figure 9:**
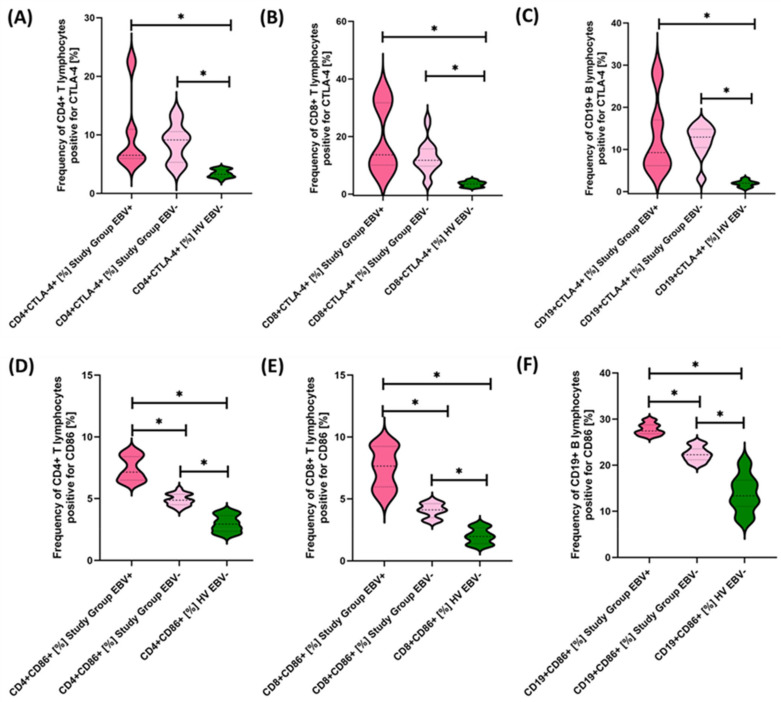
Evaluation of the percentages of T and B lymphocytes positive for CTLA-4 (**A**–**C**) and CD86 (**D**–**F**) expression in patients with antibody deficiencies with near-normal immunoglobulin levels or hyperimmunoglobulinemia, considering EBV reactivation relative to healthy volunteers. Statistically significant results are marked with *. Dark pink represents patients with antibody deficiencies with near-normal immunoglobulin levels or hyperimmunoglobulinemia showing EBV reactivation, light pink represents patients with antibody deficiencies with near-normal immunoglobulin levels or hyperimmunoglobulinemia without EBV reactivation, and green indicates the group of healthy volunteers without EBV reactivation.

**Figure 10:**
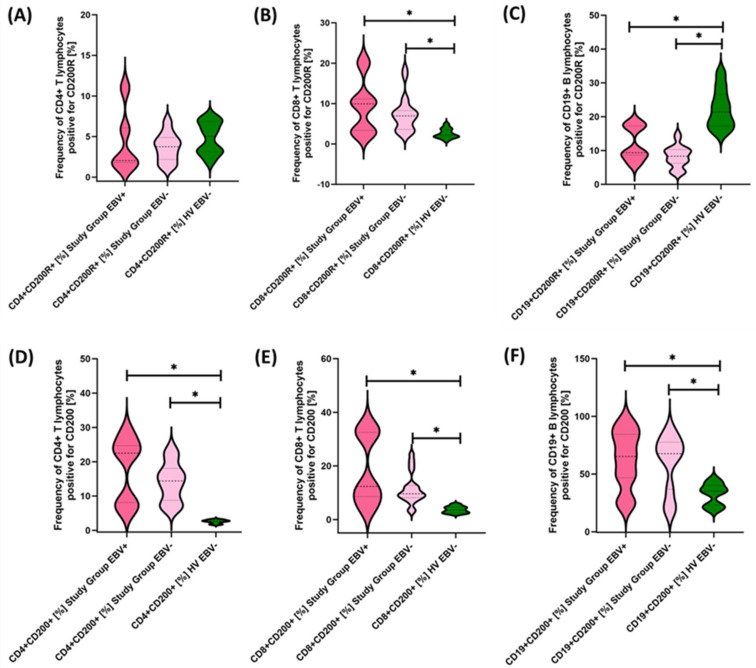
Evaluation of the percentages of CD200R (**A**–**C**) and CD200 (**D**–**F**) positive T and B lymphocytes in patients with antibody deficiencies with near-normal immunoglobulin levels or hyperimmunoglobulinemia, considering EBV reactivation relative to healthy volunteers. Statistically significant results are marked with *. Dark pink represents patients with antibody deficiencies with near-normal immunoglobulin levels or hyperimmunoglobulinemia showing EBV reactivation, light pink represents patients with antibody deficiencies with near-normal immunoglobulin levels or hyperimmunoglobulinemia without EBV reactivation, and green indicates the group of healthy volunteers without EBV reactivation.

Next, we examined in detail the results obtained from the analysis of the percentages of selected subpopulations of T and B lymphocytes positively expressing the tested molecules in the context of EBV reactivation, which is presented in Figure 8, Figure 9 and Figure 10 and summarized in Table 7.

Our analyses show that statistically significant differences between EBV+ and EBV− patients within the study group were observed for CD4+ PD-1+, CD8+ PD-1+, CD19+ PD-1, CD8+ PD-L1+, and CD19+ PD-L1+ and for CD4+ CD8+, CD8+ CD86+, and CD19+ CD86+. All these differences were higher for EBV+ than for EBV− patients. Other observed changes were not statistically significant.

**Figure 11:**
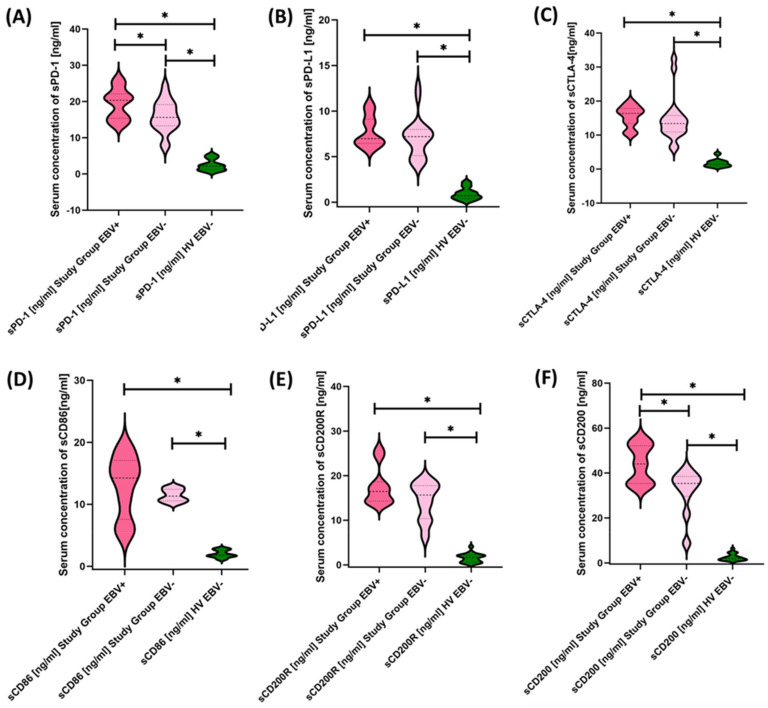
Serum Concentrations of PD-1/PD-L1 (**A**,**B**), CTLA-4/CD86 (**C**,**D**), and CD200R/CD200 (**E**,**F**) in antibody deficiencies with near-normal immunoglobulin levels or hyperimmunoglobulinemia including EBV reactivation and healthy volunteers. Statistically significant results are marked with *. Dark pink represents patients with antibody deficiencies with near-normal immunoglobulin levels or hyperimmunoglobulinemia showing EBV reactivation, light pink represents patients with antibody deficiencies with near-normal immunoglobulin levels or hyperimmunoglobulinemia without EBV reactivation, and green indicates the group of healthy volunteers without EBV reactivation.

We performed the same analysis for the values obtained from the determination of the serum concentrations of the soluble forms of the test molecules, and the results are summarized in Figure 11 and Table 8.

### 3.5. Evaluation of the Usefulness of the Tested Immune Checkpoint Pathways as a Potential Marker in Patients with Antibody Deficiencies with Near-Normal Immunoglobulin Levels or Hyperimmunoglobulinemia, Including EBV Reactivation and in Relation to Healthy Volunteers

**Figure 12:**
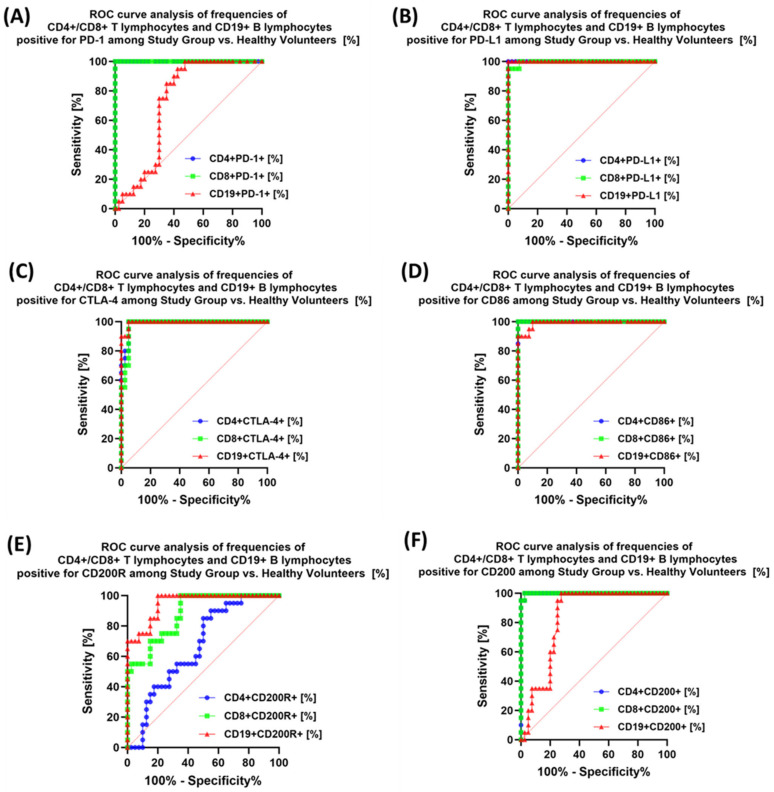
Analysis of ROC curves for the percentages of selected immune cells expressing PD-1 positive (**A**), PD-L1 (**B**), CTLA-4 (**C**), CD86 (**D**), CD200R (**E**), and CD200 (**F**) in patients with antibody deficiencies with near-normal immunoglobulin levels or hyperimmunoglobulinemia compared to healthy volunteers.

**Figure 13:**
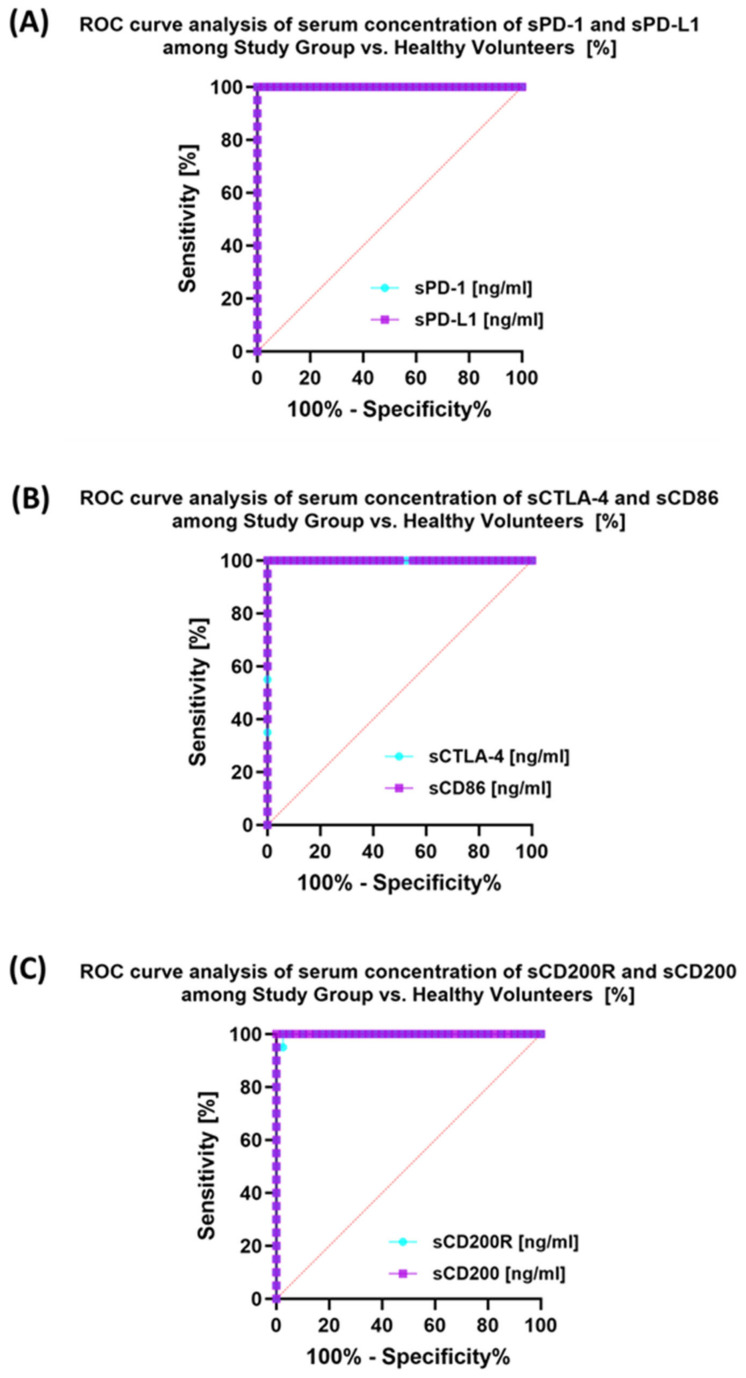
Analysis of ROC curves for serum sPD-1/sPD-L1 (**A**), sCTLA-4/sCD86 (**B**), and sCD200R/sCD200 (**C**) in patients with antibody deficiencies with near-normal immunoglobulin levels or hyperimmunoglobulinemia compared to healthy volunteers.

The last stage of the research was the sensitivity analysis of all tested parameters in terms of their usefulness as potential marker molecules, allowing for the diagnosis, monitoring, and assessment of EBV reactivation in all groups of patients. First, we analyzed using ROC curves the usefulness of the percentages of selected T and B lymphocytes positively expressing each of the tested molecules, considering the entire study group and the healthy volunteers. The results depicted in Figure 12 show that the most sensitive parameters are CD4+ PD-1+ and CD8+ PD-1+ (Figure 12A); CD4+ PD-L1+ and CD8+ PD-L1+ (Figure 12B); and CD4+ CD86+ and CD8+ CD86+ (Figure 12D). For the remaining parameters (Figure 12C,E,F), their diagnostic sensitivity was much lower. Hoveever in contrast, all analyzed concentrations of the individual soluble forms of the molecules tested also showed high sensitivity between the analyzed groups of patients (Figure 13A–C).

**Figure 14:**
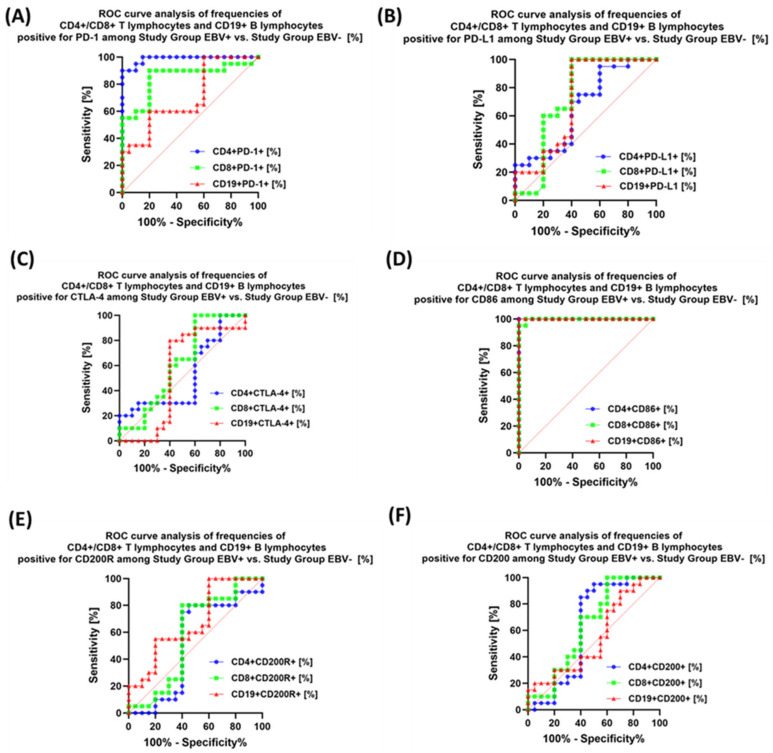
Analysis of ROC curves for the percentages of selected immune cells expressing PD-1 (**A**), PD-L1 (**B**), CTLA-4 (**C**), CD86 (**D**), CD200R (**E**), and CD200 (**F**) in patients with antibody deficiencies with near-normal immunoglobulin levels or hyperimmunoglobulinemia depending on the occurrence of EBV reactivation.

**Figure 15:**
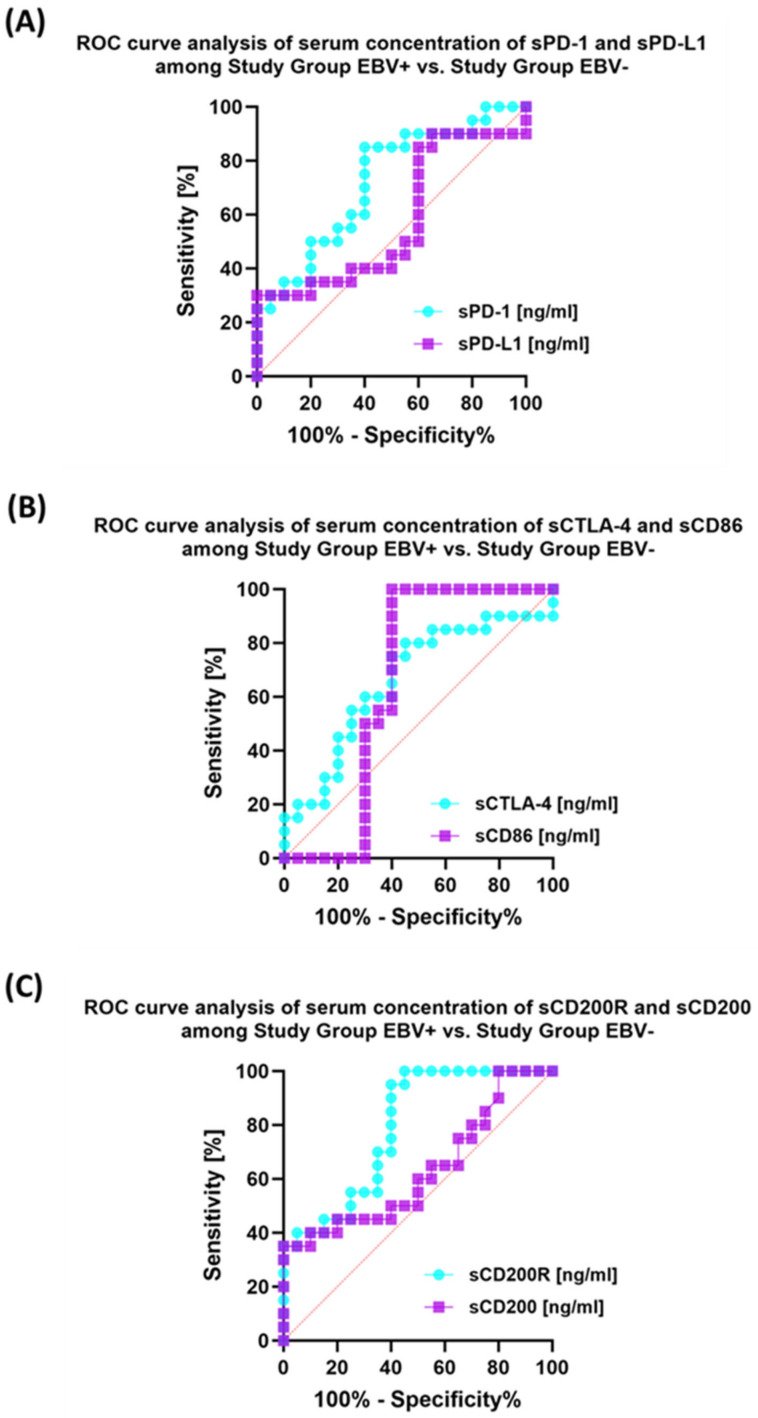
Analysis of ROC curves for serum concentrations of sPD-1/sPD-L1 (**A**), sCTLA-4/sCD86 (**B**), and sCD200R/sCD200 (**C**) in patients with antibody deficiencies with near-normal immunoglobulin levels or hyperimmunoglobulinemia depending on EBV reactivation.

Analysis of individual groups of patients with antibody deficiencies with near-normal immunoglobulin levels or hyperimmunoglobulinemia with or without EBV reactivation also showed that the most sensitive parameter was the assessment of CD86 expression on CD4+ and CD19+ (Figure 14), which may be considered potential marker molecules in the future. Unfortunately, none of the soluble molecules tested in the sera of the control patients was a sensitive parameter indicative of EBV reactivation (Figure 15).

## 4. Discussion

The results of our research presented in this manuscript are among the first reports on the role of immune checkpoints in the immunopathogenesis of antibody deficiencies with near-normal immunoglobulin levels or hyperimmunoglobulinemia. Moreover, carrying out these analyzes on newly diagnosed patients who have not yet undergone treatment sheds new light on the importance of dysregulation of the immune system in the course of the disease itself, as well as in the context of the development of PID. PID comprises a variety of inherited disorders characterized by an impaired immune response. These conditions impair the immune system’s ability to effectively fight infection, deal with malignant cells, and prevent autoimmune reactions. In people with weakened immune systems, reactivation of EBV and the persistence of EBV-infected B cells that multiply excessively are associated with serious health problems that can even lead to death. These complications include conditions such as hemophagocytic lymphohistiocytosis (HLH), also known as viral hemophagocytic syndrome, as well as benign B-cell hyperplasia (LPD) and various types of B-cell lymphomas, such as Hodgkin’s lymphoma, Burkitt’s lymphoma, and diffuse large cell lymphoma B cell (DLBCL) [34].

Moreover, our team’s observed involvement (reactivation) of oncogenic EBV in antibody deficiencies with near-normal immunoglobulin levels or hyperimmunenoglobulinemia, as well as interactions with tested immune checkpoints, offering insight into a small part of the complex immune dysregulation observed in PID.

In the context of EBV, PIDs show a diverse spectrum of responses, often leading not only to disturbances in the functioning of the immune response but also to the development of cancer. Although this topic is extremely important in the literature, one can find only a dozen or so articles discussing this issue in various diseases classified as PID, including: activated phosphatidylinositide 3-kinase delta (PI3Kδ) syndrome (APDS) [35,36,37]; LPS-responsive beige-like anchor (LRBA) deficiency [38,39]; autoimmune lymphoproliferative syndrome (ALPS) [40,41,42]; familial HLH (FHL) [43]; ZAP70 deficiency [44]; tyrosine kinase 2 (TYK2) [45]; and Wiskott–Aldrich syndrome (WAS) [46,47,48].

Over the past two decades, our knowledge of host–virus interactions in EBV infection has improved significantly. This knowledge has led to a better understanding of the mechanisms underlying severe and atypical EBV infections. By identifying new genes associated with EBV-related immunodeficiency and disease susceptibility, we are gradually uncovering the cellular, biochemical, and molecular processes that regulate EBV resistance.

Immune checkpoints, including molecules such as PD-1/PD-L1, CTLA-4/CD86, and CD200R/CD200, play key roles in regulating immune responses. In PID, dysfunctional expression of the immune checkpoint disrupts immune homeostasis, affecting both antiviral defense mechanisms and immune tolerance, but detailed research on this extremely important aspect remains scarce [49]. The interaction between EBV and immune checkpoints is particularly evident in PID. Some PIDs show altered patterns of immune checkpoint expression on T and B lymphocytes during EBV infection, which is also observed in our study results. This imbalance contributes to impaired immune responses, resulting in chronic EBV reactivation and susceptibility to EBV-related cancers, especially lymphomas [50,51,52].

The research results presented in this manuscript, although extremely interesting because they were found in newly diagnosed patients, also have their limitations. First, they show only a small part of the changes that occur in the immune system in patients with antibody deficiencies with near-normal immunoglobulin levels or hyperimmunoglobulinemia and interactions related to the reactivation of the EBV virus. Additionally, the sample size was not large enough to draw clear conclusions regarding EBV infection and immunological checkpoints in the development of cancer in the studied patients. However, we hope that the presented research results will contribute to increasing the interest of researchers and physicians, as well as to an increased number of interdisciplinary studies allowing for a better understanding of the observed immune deregulations. Understanding the complex relationships among EBV, immune checkpoints, and PID is crucial for tailored therapeutic interventions. Diagnosing and treating these PIDs requires specialized medical care from immunologists, allergists, and other health care professionals. Treatment strategies may include immunoglobulin replacement therapy, treating infections, and addressing underlying immune system dysfunctions to improve the overall health and quality of life of people with these conditions.

Additionally, chronic or recurrent EBV reactivation may contribute to immune system dysregulation and increase the risk of EBV-related complications, including certain types of lymphoma, particularly in the setting of long-term immune system dysfunction. Therefore, monitoring, appropriate treatment, and an approach tailored to an individual’s specific health situation are essential to optimize immune function and minimize potential complications. It is important to note that, although some primary immunodeficiencies may increase the risk of serious complications from EBV, not all people with primary immunodeficiencies will have the same susceptibility. Additionally, the impact of EBV on people with PID can vary greatly depending on specific genetic mutations and the resulting immune system dysfunction. Targeting immune system checkpoints can rebalance the immune system by enhancing antiviral responses and reducing EBV-related complications. Advances in this area have the potential to produce innovative treatments that will change the trajectory of PID, improving patient outcomes and quality of life.

## 5. Conclusions

The research results presented in the article constitute only a small part of the immune deregulations occurring in the course of this group of diseases, and due to the relatively small group of patients, our conclusions are only suggestions and require further interdisciplinary research to confirm the observed changes.

Understanding the complex connections among immune checkpoints, antibody deficiencies, and EBV reactivation is essential for the development of targeted therapeutic approaches. Modulating immune checkpoints may offer new opportunities to enhance antibody responses and reduce the risk of EBV-related infections and complications. We hope that, as research in this field progresses, researchers, in collaboration with physicians, will revolutionize the treatment of antibody deficiencies with near-normal immunoglobulin levels or hyperimmunoglobulinemia in the near future, ultimately improving patient outcomes and reducing the risk of developing cancer. 

## Figures and Tables

**Table 1:** Selected blood morphology and biochemistry results of patients with antibody deficiencies with near-normal immunoglobulin levels or hyperimmunoglobulinemia compared to healthy volunteers.

Parameter	Study Group		Healthy Volunteers		*p*-Value
	Mean ± SD	Median (Range)	Mean ± SD	Median (Range)	
WBC	7.75 ± 1.33	7.65 (5.03–9.99)	6.15 ± 0.96	6.14 (4.75–7.73)	0.000 *
LYM	2.10 ± 0.7	2.03 (1.03–3.96)	1.84 ± 0.32	1.85 (1.23–2.76)	0.181
MON	0.43 ± 0.11	0.45 (0.20–0.61)	0.47 ± 0.11	0.48 (0.28–0.63)	0.176
NEU	4.32 ± 1.38	4.92 (2.08–6.18)	3.7 ± 1.07	3.48 (1.83–5.44)	0.070
RBC	3.98 ± 0.66	4.07 (2.54–5.10)	4.56 ± 0.31	4.57 (3.95–5.10)	0.000 *
HGB	12.97 ± 2.32	13.47 (8.25–16.05)	13.48 ± 1.28	13.30 (11.80–15.50)	0.797
PLT	213.53 ± 59.99	212.82 (93.28–351.00)	243.85 ± 51.21	231.50 (155.00–346.00)	0.052
IgG	14.55 ± 2.34	14.78 (9.86–18.92)	13.1 ±1 1.66	12.85 (10.16–15.94)	0.020 *
IgM	1.49 ± 0.56	1.45 (0.55–2.63)	1.31 ± 0.51	1.17 (0.60–2.21)	0.203
IgA	1.76 ± 0.73	1.85 (0.59–3.18)	2.05 ± 0.80	1.84 (0.70–4.00)	0.353

* statistically significant results.

**Table 2:** Changes in the immunophenotype of patients with antibody deficiencies with near-normal immunoglobulin levels or hyperimmunoglobulinemia compared to healthy volunteers.

Parameter	Study Group		Healthy Volunteers		*p*-Value
	Mean ± SD	Median (Range)	Mean ± SD	Median (Range)	
CD45+ [%]	91.08 ± 4.31	91.35 (82.29–97.33)	93.97 ± 2.35	94.38 (90.38–97.91)	0.147
CD3+ [%]	65.76 ± 11.38	67.53 (40.84–89.89)	74.32 ± 5.61	73.61 (67.20–94.58)	0.000 *
CD19+ [%]	9.90 ± 5.82	9.58 (2.56–24.22)	13.29 ± 1.74	12.76 (11.05–16.82)	0.000 *
CD4+ [%]	34.23 ± 19.36	29.19 (11.38–67.92)	48.35 ± 4.53	47.49 (42.25–61.31)	0.000 *
CD8+ [%]	30.03 ± 15.21	25.88 (11.49–57.29)	27.24 ± 2.31	27.30 (22.25–31.07)	0.602
CD4+/CD8+ ratio	1.48 ± 1.32	1.02 (0.25–5.08)	1.79 ± 0.21	1.78 (1.53–2.13)	0.001 *

* statistically significant results.

**Table 3:** Percentage of tested immune cells positively expressing tested immune checkpoint pathways.

Parameter		Study Group		Healthy Volunteers		*p*-Value
		Mean ± SD	Median (Range)	Mean ± SD	Median (Range)	
PD-1	CD4+ PD-1+	15.38 ± 5.16	15.29 (6.52–27.72)	3.32 ± 1.18	3.00 (1.18–5.68)	0.000 *
	CD8+ PD-1+	20.38 ± 9.74	17.41 (5.17–37.47)	2.39 ± 1.09	2.15 (0.79–4.28)	0.000 *
	CD19+ PD-1+	7.68 ± 4.98	6.92 (2.01–17.59)	4.31 ± 1.42	4.22 (1.97–6.82)	0.004 *
PD-L1	CD4+ PD-L1+	8.37 ± 3.49	7.51 (2.55–13.92)	0.82 ± 0.33	0.87 (0.21–1.33)	0.000 *
	CD8+ PD-L1+	7.35 ± 4.62	6.36 (0.97–17.00)	0.55 ± 0.29	0.57 (0.01–1.01)	0.000 *
	CD19+ PD-L1+	9.03 ± 4.84	7.37 (1.62–17.87)	0.28 ± 0.13	0.28 (0.03–0.48)	0.000 *
CTLA-4	CD4+ CTLA-4+	9.44 ± 5.11	8.29 (4.17–23.55)	3.39 ± 0.66	3.27 (2.18–4.41)	0.000 *
	CD8+ CTLA-4+	16.09 ± 9.52	12.89 (4.16–36.89)	3.51 ± 0.90	3.52 (2.10–5.12)	0.000 *
	CD19+ CTLA-4+	12.57 ± 6.58	11.97 (3.12–29.42)	1.88 ± 0.70	1.93 (0.59–3.17)	0.000 *
CD86	CD4+ CD86+	6.17 ± 1.48	6.03 (4.08–8.89)	2.97 ± 0.65	2.94 (2.02–3.97)	0.000 *
	CD8+ CD86+	5.78 ± 2.09	4.85 (3.14–9.90)	1.98 ± 0.60	1.96 (1.13–3.00)	0.000 *
	CD19+ CD86+	25.11 ± 3.04	25.59 (20.05–29.88)	13.80 ± 3.86	13.36 (8.03–20.89)	0.000 *
CD200R	CD4+ CD200R+	4.04 ± 2.89	3.13 (1.13–11.52)	4.84 ± 1.88	5.04 (1.89–7.44)	0.000 *
	CD8+ CD200R +	8.33 ± 5.49	7.53 (2.25–21.02)	2.58 ± 1.16	2.24 ?(0.74–5.06)	0.003 *
	CD19+ CD200R +	10.06 ± 4.40	9.22 (3.51–18.41)	22.35 ± 5.56	21.39 (15.27–33.00)	0.000 *
CD200	CD4+ CD200+	15.98 ± 7.61	15.82 (5.80–29.38)	2.58 ± 0.51	2.70 (1.58–3.30)	0.000 *
	CD8+ CD200+	14.28 ± 10.11	10.96 (3.21–35.29)	3.67 ± 1.19	3.58 (1.88–5.81)	0.000 *
	CD19+ CD200+	61.06 ± 23.84	67.50 (16.83–96.82)	32.64 ± 9.09	35.13 (18.15–46.58)	0.000 *

* statistically significant results.

**Table 4:** Serum concentrations of PD-1/PD-L1, CTLA-4/CD86, and CD200R/CD200 in patients with antibody deficiencies with near-normal immunoglobulin levels or hyperimmunoglobulinemia relative to healthy volunteers.

Serum Concentration [ng/mL]	Study Group		Healthy Volunteers		*p*-Value
	Mean ± SD	Median (Range)	Mean ± SD	Median (Range)	
sPD-1	17.70 ± 4.52	17.09 (7.53–26.49)	2.44 ± 1.52	2.19 (0.52–5.42)	0.000 *
sPD-L1	7.41 ± 1.95	7.08 (4.35–12.73)	0.92 ± 0.63	0.71 (01.0–2.50)	0.000 *
sCTLA-4	14.96 ± 4.93	14.64 (6.75–32.53)	1.73 ± 1.14	1.45 (0.40–4.74)	0.000 *
sCD86	12.19 ± 3.52	11.89 (4.94–19.71)	1.99 ± 0.58	1.82 (1.06–2.91)	0.000 *
sCD200R	15.92 ± 4.40	16.24 (6.12–26.22)	2.48 ± 1.49	2.00 (0.89–6.29)	0.002 *
sCD200	38.09 ± 10.64	37.12 (8.34–55.46)	1.55 ± 0.97	1.67 (0.11–4.08)	0.000 *

* statistically significant results.

**Table 5:** Specific anti-EBV antibody serum concentration.

Antibody Serum Concentration [U/mL]		Study Group				HV			*p*-Value		
		EBV+ (Group 1)		EBV− (Group 2)		EBV− (Group 3)					
		Mean ± SD	Median (Range)	Mean ± SD	Median (Range)	Mean ± SD	Median (Range)	All Groups	1 vs. 2	1 vs. 3	2 vs. 3
Anti-EBV EA	IgA	46.46 ± 10.22	44.68 (31.76–69.49)	4.84 ± 1.14	4.61 (3.07–6.70)	4.89 ± 1.59	5.31 (2.29–6.96)	0.000 *	0.000 *	0.000 *	0.778
	IgM	6.68 ± 1.71	6.49 (4.03–9.20)	4.88 ± 0.50	4.75 (4.13–5.85)	4.98 ± 1.03	4.82 (3.15–6.98)	0.000 *	0.000 *	0.003 *	0.639
	IgG	74.30 ± 11.78	74.98 (52.67–91.16)	4.21 ± 1.08	4.29 (2.20–5.95)	3.68 ± 1.17	3.24 (2.16–5.62)	0.000 *	0.000 *	0.000 *	0.201
Anti-EBV VCA	IgA	16.79 ± 2.81	16.77 (12.50–21.17)	2.94 ± 1.10	2.91 (1.05–4.91)	5.04 ± 0.92	4.92 (3.47–6.80)	0.000 *	0.000 *	0.000 *	0.000 *
	IgM	4.23 ± 8.55	44.63 (26.01–56.58)	4.57 ± 1.67	4.48 (2.22–6.96)	5.63 ± 1.87	6.06 (2.59–8.83)	0.000 *	0.000 *	0.000 *	0.067
	IgG	167.76 ± 23.44	158.67 (132.67–209.87)	154.17 ± 23.34	160.77 (113.07–189.16)	112.29 ± 22.97	118.41 (76.58–151.11)	0.000 *	0.191	0.000 *	0.000 *
Anti-EBV EBNA-1	IgA	13.75 ± 1.07	13.79 (12.24–15.77)	2.92 ± 1.03	3.28 (1.02–4.62)	3.34 ± 1.39	2.97 (1.22–5.65)	0.000 *	0.000 *	0.000 *	0.429
	IgM	6.96 ± 2.51	7.04 (2.73–10.63)	3.98 ± 1.00	4.12 (2.47–5.42)	5.34 ± 1.42	5.26 (3.22–7.96)	0.000 *	0.000 *	0.04 *	0.005 *
	IgG	235.16 ± 16.86	240.70 (207.77–259.26)	67.58 ± 7.20	67.82 (52.78–79.56)	62.65 ± 10.92	61.17 (45.62–78.50)	0.000 *	0.000 *	0.000 *	0.231

* statistically significant results.

**Table 6:** Comparative analysis of selected peripheral blood parameters of patients, including EBV reactivation.

Parameter	Study Group				HV		*p*-Value			
	EBV+ (Group 1)		EBV− (Group 2)		EBV− (Group 3)					
	Mean ± SD	Median (Range)	Mean ± SD	Median (Range)	Mean ± SD	Median (Range)	All Groups	1 vs. 2	1 vs. 3	2 vs. 3
WBC	7.31 ± 1.19	7.57 (5.03–8.99)	8.18 ± 1.32	7.98 (5.90–9.99)	6.15 ± 0.96	6.14 (4.75–7.73)	0.000 *	0.052	0.002 *	0.000 *
LYM	1.61 ± 0.40	1.56 (1.03–2.57)	2.59 ± 0.61	2.54 (1.45–3.96)	1.84 ± 0.32	1.85 (1.23–2.76)	0.181	0.000 *	0.067	0.000 *
MON	0.40 ± 0.10	0.41 (0.20–0.52)	0.46 ± 0.10	0.48 (0.25–0.61)	0.47 ± 0.11	0.48 (0.28–0.63)	0.176	0.063	0.037 *	0.799
NEU	4.41 ± 1.49	5.13 (2.16–6.18)	4.23 ± 1.26	4.72 (2.08–5.98)	3.7 ± 1.07	3.48 (1.83–5.44)	0.070	0.231	0.114	0.140
RBC	3.61 ± 0.68	3.53 (2.54–5.10)	4.35 ± 0.36	4.33 (3.79–5.10)	4.56 ± 0.31	4.57 (3.95–5.10)	0.000 *	0.000 *	0.000 *	0.049 *
HGB	11.34 ± 2.11	11.22 (8.25–15.70)	14.61 ± 1.02	14.79 (12.14–16.05)	13.48 ± 1.28	13.30 (11.80–15.50)	0.797	0.000 *	0.001 *	0.006 *
PLT	176.06 ± 48.09	172.03 (93.28–292.00)	251.00 ± 45.58	233.62 (196.73–351.00)	243.85 ± 51.21	231.50 (155.00–346.00)	0.052	0.000 *	0.000 *	0.758
IgG	15.95 ± 1.38	15.62 (13.97–18.92)	13.14 ± 2.25	12.89 (9.86–16.83)	13.11 ± 1.66	12.85 (10.16–15.94)	0.020 *	0.000 *	0.000 *	0.883
IgM	1.61 ± 0.56	1.61 (0.74–2.58)	1.38 ± 0.53	1.33 (0.55–2.63)	1.31 ± 0.51	1.17 (0.60–2.21)	0.203	0.253	0.096	0.601
IgA	1.83 ± 0.67	1.91 (0.59–3.18)	1.69 ± 0.77	1.5 (0.71–3.03)	2.05 ± 0.80	1.84 (0.70–4.00)	0.353	0.698	0.601	0.298
CD3+ [%]	62.00 ± 9.05	62.34 (40.84–78.49)	69.53 ± 12.20	71.03 (46.43–89.89)	74.32 ± 5.61	73.61 (67.20–94.58)	0.000 *	0.013 *	0.000 *	0.141
CD19+ [%]	7.09 ± 2.46	7.46 (2.65–10.63)	12.72 ± 6.77	11.81 (2.56–24.22)	13.29 ± 1.74	12.76 (11.05–16.82)	0.000 *	0.002 *	0.000 *	0.327
CD4+ [%]	26.29 ± 9.70	24.98 (11.38–43.29)	42.16 ± 21.31	46.59 (14.09–67.92)	48.35 ± 4.53	47.49 (42.25–61.31)	0.000 *	0.040 *	0.000 *	0.738
CD8+ [%]	25.98 ± 12.08	26.51 (11.49–54.43)	34.08 ± 16.85	46.59 (14.09–67.92)	27.24 ± 2.31	27.30 (22.25–31.07)	0.672	0.368	0.564	0.758
CD4+/CD8+ ratio	1.20 ± 0.83	0.87 (0.37–3.38)	1.76 ± 1.62	1.13 (0.25–5.08)	1.79 ± 0.21	1.78 (1.53–2.13)	0.001 *	0.461	0.000 *	0.045 *

* statistically significant results.

**Table 7:** Comparative analysis of the impact of EBV reactivation on the expression of the tested molecules on selected subpopulations of T and B lymphocytes in patients with antibody deficiencies with near-normal immunoglobulin levels or hyperimmunoglobulinemia and healthy volunteers.

Parameter		Study Group				HV		*p*-Value			
		EBV+ (Group 1)		EBV− (Group 2)		EBV− (Group 3)					
		Mean ± SD	Median (Range)	Mean ± SD	Median (Range)	Mean ± SD	Median (Range)	All Groups	1 vs. 2	1 vs. 3	2 vs. 3
PD-1	CD4+ PD-1+	19.31 ± 3.34	17.81 (14.83–24.72)	11.44 ± 3.33	13.11 (5.93–16.02)	3.32 ± 1.18	3.00 (1.18–5.68)	0.000 *	0.000 *	0.000 *	0.000 *
	CD8+ PD-1+	26.0 ± 7.78	25.39 (14.33–37.47)	14.76 ± 8.11	13.85 (7.70–37.47)	2.39 ± 1.09	2.15 (0.79–4.28)	0.000 *	0.000 *	0.000 *	0.000 *
	CD19+ PD-1+	10.11 ± 5.67	7.13 (2.93–17.59)	5.26 ± 2.39	5.69 (1.83–8.32)	4.31 ± 1.42	4.22 (1.97–6.82)	0.000 *	0.022 *	0.000 *	0.242
PD-L1	CD4+ PD-L1+	9.55 ± 3.43	9.74 (5.01–13.92)	7.20 ± 3.14	7.07 (2.32–13.35)	0.82 ± 0.33	0.87 (0.21–1.33)	0.000 *	0.08	0.000 *	0.000 *
	CD8+ PD-L1+	9.43 ± 5.34	10.82 (0.97–17.00)	5.27 ± 2.34	4.87 (0.96–9.16)	0.55 ± 0.29	0.57 (0.01–1.01)	0.000 *	0.009 *	0.000 *	0.000 *
	CD19+ PD-L1+	11.44 ± 5.27	13.18 (4.44–17.87)	6.61 ± 2.73	6.52 (1.47–11.19)	0.28 ± 0.13	0.28 (0.03–0.48)	0.000 *	0.001 *	0.000 *	0.000 *
CTLA-4	CD4+ CTLA-4+	10.20 ± 6.43	6.52 (5.04–23.55)	8.67 ± 3.11	9.13 (3.79–14.09)	3.39 ± 0.66	3.27 (2.18–4.41)	0.000 *	0.841	0.000 *	0.000 *
	CD8+ CTLA-4+	19.38 ± 11.38	13.65 (6.64–36.89)	12.80 ± 5.50	11.71 (3.79–26.43)	3.51 ± 0.90	3.52 (2.10–5.12)	0.000 *	0.231	0.000 *	0.000 *
	CD19+ CTLA-4+	13.07 ± 8.49	9.25 (4.96–29.42)	12.07 ± 3.74	12.93 (2.84–16.40)	1.88 ± 0.70	1.93 (0.59–3.17)	0.000 *	0.698	0.000 *	0.000 *
CD86	CD4+ CD86+	7.45 ± 0.96	7.14 (6.24–8.89)	4.90 ± 0.46	4.87 (4.08–5.81)	2.97 ± 0.65	2.94 (2.02–3.97)	0.000 *	0.000 *	0.000 *	0.000 *
	CD8+ CD86+	7.50 ± 1.60	7.65 (4.75–9.90)	4.07 ± 0.56	4.11 (3.14–4.94)	1.98 ±0.60	1.96 (1.13–3.00)	0.000 *	0.000 *	0.000 *	0.000 *
	CD19+ CD86+	27.82 ± 1.20	27.40 (26.25–29.88)	22.39 ± 1.53	22.26 (20.05–24.93)	13.80 ± 3.86	13.36 (8.03–20.89)	0.000 *	0.000 *	0.000 *	0.000 *
CD200R	CD4+ CD200R+	4.42 ± 3.67	2.04 (1.34–11.52)	3.66 ± 1.70	3.75 (1.03–7.13)	4.84 ± 1.88	5.04 (1.89–7.44)	0.121	0.841	0.091	0.067
	CD8+ CD200R +	9.33 ± 6.35	9.92 (2.25–21.02)	7.34 ± 4.23	6.96 (2.24–18.51)	2.58 ± 1.16	2.24 (0.74–5.06)	0.000 *	0.383	0.000 *	0.000 *
	CD19+ CD200R +	11.90 ± 4.72	9.38 (5.98–18.41)	8.22 ± 3.12	8.32 (3.19–14.93)	22.35 ± 5.56	21.39 (15.27–33.00)	0.000 *	0.055	0.000 *	0.000 *
CD200	CD4+ CD200+	17.81 ± 8.86	22.50 (5.80–29.38)	14.14 ± 5.53	14.41 (5.81–24.28)	2.58 ± 0.51	2.70 (1.58–3.30)	0.000 *	0.173	0.000 *	0.000 *
	CD8+ CD200+	18.34 ± 12.21	12.37 (4.34–35.29)	11.42 ± 5.62	9.62 (3.21–24.43)	3.67 ± 1.19	3.58 (1.88–5.81)	0.000 *	0.141	0.000 *	0.000 *
	CD19+ CD200+	62.54 ± 24.06	65.21 (23.83–96.82)	59.59 ± 23.52	67.69 (16.83–90.26)	32.64 ± 9.09	35.13 (18.15–46.58)	0.000 *	0.601	0.000 *	0.000 *

* statistically significant results.

**Table 8:** Serum concentrations of PD-1/PD-L1 (Figure 11A,B), CTLA-4/CD86 (Figure 11C,D), and CD200R/CD200 (Figure 11E,F) in antibody deficiencies with near-normal immunoglobulin levels or hyperimmunoglobulinemia including EBV reactivation and healthy volunteers.

Serum Concentration [ng/mL]	Study Group				HV		*p*-Value			
	EBV+ (Group 1)		EBV− (Group 2)		EBV− (Group 3)					
	Mean ± SD	Median (Range)	Mean ± SD	Median (Range)	Mean ± SD	Median (Range)	All Groups	1 vs. 2	1 vs. 3	2 vs. 3
sPD-1	19.43 ± 4.11	20.34 (13.28–26.49)	15.96 ± 4.23	15.61 (7.53–24.53)	2.44 ± 1.52	2.19 (0.52–5.42)	0.000 *	0.016 *	0.000 *	0.000 *
sPD-L1	7.74 ± 1.64	6.98 (5.72–10.89)	7.08 ± 2.16	7.20 (4.06–12.73)	0.92 ± 0.63	0.71 (01.0–2.50)	0.000 *	0.410	0.000 *	0.000 *
sCTLA-4	15.48 ± 2.99	16.36 (10.11–19.96)	14.43 ± 6.25	13.39 (6.14–32.53)	1.73 ± 1.14	1.45 (0.40–4.74)	0.000 *	0.076	0.000 *	0.000 *
sCD86	12.94 ± 4.77	14.24 (4.34–19.71)	11.44 ± 0.97	11.32 (10.00–12.93)	1.99 ± 0.58	1.82 (1.06–2.91)	0.000 *	0.102	0.000 *	0.000 *
sCD200R	17.53 ± 4.09	16.45 (13.43–26.22)	14.30 ± 4.10	14.63 (6.12–19.09)	2.48 ± 1.49	2.00 (0.89–6.29)	0.000 *	0.157	0.002 *	0.002 *
sCD200	44.01 ± 8.14	44.06 (32.85–55.46)	32.17 ± 9.49	35.36 (8.74–42.78)	1.55 ± 0.97	1.67 (0.11–4.08)	0.000 *	0.001 *	0.000 *	0.000 *

* statistically significant results.

## Data Availability

All necessary information regarding the preparation of this work is available by written request to the corresponding author.
